# Cancer-associated noncoding mutations affect RNA G-quadruplex-mediated regulation of gene expression

**DOI:** 10.1038/s41598-017-00739-y

**Published:** 2017-04-06

**Authors:** Mahdi Zeraati, Aaron L. Moye, Jason W. H. Wong, Dilmi Perera, Mark J. Cowley, Daniel U. Christ, Tracy M. Bryan, Marcel E. Dinger

**Affiliations:** 1 Garvan Institute of Medical Research, Sydney, NSW 2010 Australia; 2 St Vincent’s Clinical School, Faculty of Medicine, University of New South Wales, Sydney, NSW 2052 Australia; 3 Children’s Medical Research Institute, University of Sydney, Sydney, NSW 2145 Australia; 4 Prince of Wales Clinical School, Faculty of Medicine, University of New South Wales, Sydney, NSW 2052 Australia

## Abstract

Cancer is a multifactorial disease driven by a combination of genetic and environmental factors. Many cancer driver mutations have been characterised in protein-coding regions of the genome. However, mutations in noncoding regions associated with cancer have been less investigated. G-quadruplex (G4) nucleic acids are four-stranded secondary structures formed in guanine-rich sequences and prevalent in the regulatory regions. In this study, we used published whole cancer genome sequence data to find mutations in cancer patients that overlap potential RNA G4-forming sequences in 5′ UTRs. Using RNAfold, we assessed the effect of these mutations on the thermodynamic stability of predicted RNA G4s in the context of full-length 5′ UTRs. Of the 217 identified mutations, we found that 33 are predicted to destabilise and 21 predicted to stabilise potential RNA G4s. We experimentally validated the effect of destabilising mutations in the 5′ UTRs of *BCL2* and *CXCL14* and one stabilising mutation in the 5′ UTR of *TAOK2*. These mutations resulted in an increase or a decrease in translation of these mRNAs, respectively. These findings suggest that mutations that modulate the G4 stability in the noncoding regions could act as cancer driver mutations, which present an opportunity for early cancer diagnosis using individual sequencing information.

## Introduction

Substantial efforts have been made to characterise the cancer genome. The advent of next generation sequencing has enabled affordable and rapid large-scale sequencing of cancer genomes. The Cancer Genome Atlas (TCGA)^1^ and the International Cancer Genome Consortium^2^ are examples of large-scale cancer genomics projects that aim to catalogue cancer-associated mutations. One of the main goals of these projects is to differentiate cancer driver mutations, which contribute to cancer development, from non-functional passenger mutations and ultimately improve our understanding of how different types of cancers develop^3^. As a result of extensive efforts that have been directed towards detecting and characterising driver mutations in protein-coding regions, knowledge about abnormal proteins encoded by mutated cancer genes has grown substantially in recent years, such that new cancer therapeutics have been developed to target these anomalous proteins. Imatinib is an example of this type of therapeutic that inhibits the protein encoded by *ABL*, which is mutated in chronic myeloid leukaemia^4^. Noncoding somatic mutations, on the other hand, have not been examined as extensively as coding variations. Considering that less than 2% of the human genome encodes protein and that important regulatory regions including promoters and 5′ and 3′ untranslated regions (UTR) of mRNAs are located in the noncoding regions^5^, effective functional studies of mutations in the noncoding regions would provide a better understanding of cancer biology and development. Indeed, several studies have indicated that noncoding somatic mutations can alter gene expression in cancer^6–8^, prompting the hypothesis that these mutations affect regulatory motifs.

Guanine (G)-rich DNA and RNA sequences can fold into non-canonical secondary structures called G-quadruplexes (G4s). The building block of a G4 is called a G-tetrad, which consists of four guanines in a square planar arrangement. Each guanine is connected to the two adjacent guanines by hydrogen bonds at the Watson Crick and Hoogsteen edges. The G4 structure is formed by at least two stacked G-tetrads. A cation positioned at the center of each tetrad or between them further stabilises the G4 structure. Nucleotides that do not contribute to the G-tetrads form the loops^9^. Intramolecular G4 is assembled from guanines within one nucleic acid strand, whereas separate nucleic acid strands can form an intermolecular G4. Intramolecular DNA G4s can adopt various topologies^10^, whereas intramolecular RNA G4s predominantly fold into the parallel propeller topology^11^. Several different G4 structures have been studied *in vitro*. The results from these studies have enabled the development of algorithms that predict potential G4-forming sequences in the genome^12–14^. Using these algorithms, computational studies have demonstrated that G4s are significantly enriched and conserved in regulatory elements, such as promoters and UTRs of mRNAs^15^. This observation suggests that G4s may have regulatory roles and be involved in biological processes.

A number of studies have demonstrated that G4s located in the 5′ UTRs modulate translation activity. Although it has been shown that G4 can augment translation^16^, most G4s that have been characterised to date act as repressors in the cap-dependent translation regulation of mRNA translation^17–20^, especially in case of proto-oncogenes, such as *NRAS* ^21^. Given accumulating *in vitro* results that demonstrate 5′ UTR G4s have regulatory functions and the increasing evidence that show G4 structures exist *in vivo* ^22, 23^ and a number are altered in cancer tissues^24^, we hypothesized that mutations in cancer patients alter the stability of 5′ UTR G4s and consequently affect their regulatory function.

Here, we present an approach that permitted us to identify somatic mutations in cancer that change the stability of 5′ UTR G4s. We have experimentally validated the effect of some of these mutations and showed that they can either destabilise RNA G4 structures or stabilise them. Furthermore, the mutations result in altered gene expression. Overall, our data suggest that mutations in regulatory elements such as 5′ UTR that alter G4s stability could act as cancer driver mutations.

## Results

### Somatic mutations in cancer patients change the thermodynamic stability of RNA G4s in the 5′ UTR

**Figure 1:**
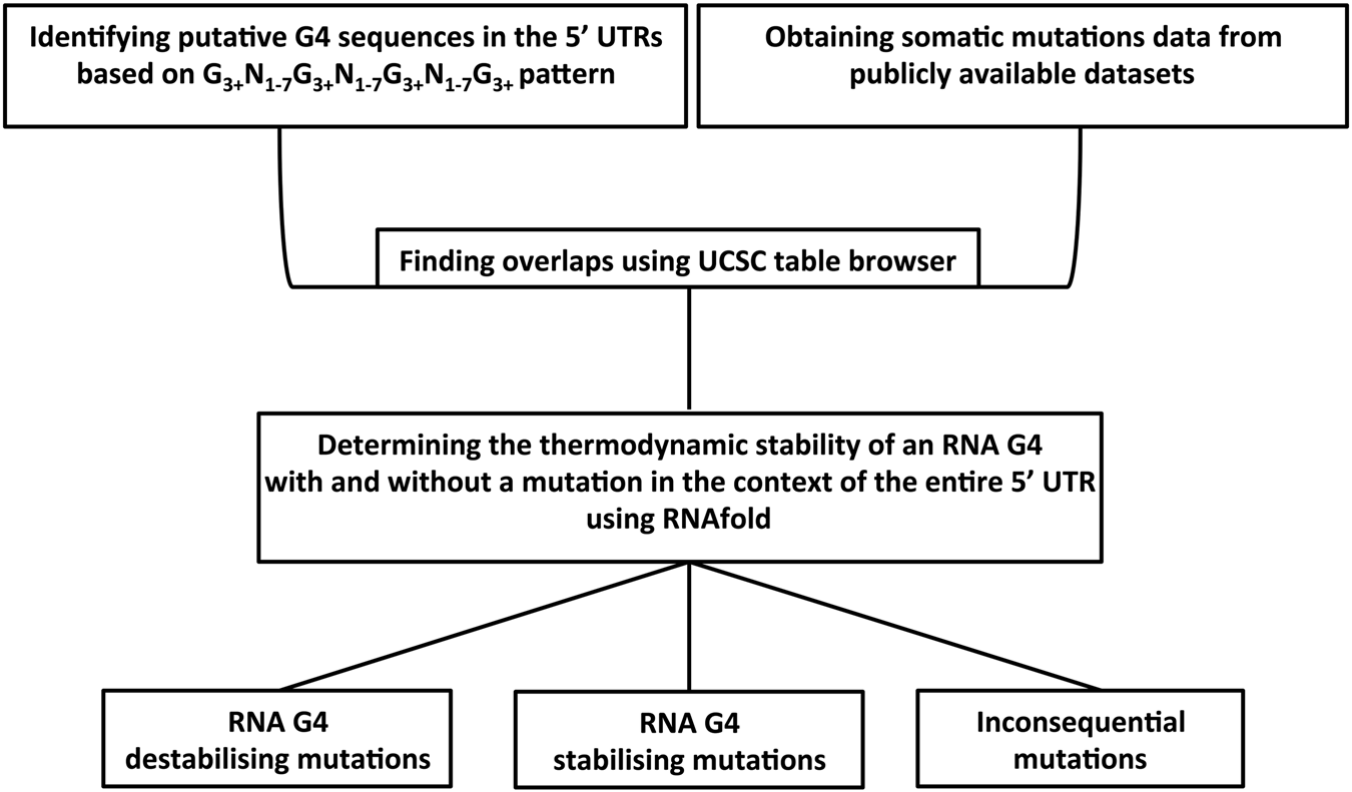
Flowchart illustrating the procedure for identifying mutations that change predicted RNA G4 stability in the 5′ UTRs of genes mutated in cancer.

Previous studies have demonstrated that RNA G4s act as regulatory motifs in the 5′ UTR of mRNAs. To determine the possible effects of mutations in cancer patients on the stability of G4s in this region, the procedure illustrated in Fig. 1 was followed. A dataset of potential G4-forming sequences in the human genome was constructed. Quadparser algorithm^13^, which searches for the sequence pattern G_3+_N_1–7_G_3+_N_1–7_G_3+_N_1–7_G_3+_ across the genome, was employed to identify potential G4s. In this pattern, G refers to guanine and N can be any base including guanine. Concurrently, three publicly available databases, TCGA^1^, ICGC^2^ and Alexandrov *et al*.^25^, were used to obtain somatic mutation data in patients with different types of cancers. Comparing these two datasets, 217 overlaps were found between somatic mutations from cancer patients and potential G4-forming sequences in the 5′ UTRs (Supplementary Dataset). Considering that putative G4 sequences might fold into other secondary structures instead of G4 structures, it is possible that overlapping mutations do not affect RNA G4 structural stability. To address this possibility, a further screening step was performed that investigated the effect of each mutation on the predicted RNA G4 stability using RNAfold software^26^. In addition to the G4 sequence pattern (G_2–7_N_1–15_G_2–7_N_1–15_G_2–7_N_1–15_G_2–7_), RNAfold takes account of thermodynamic parameters and is therefore a more stringent method to predict G4 structures. To simulate a more biologically relevant condition, the effect of each mutation was examined in the context of the entire 5′ UTR.

**Figure 2:**
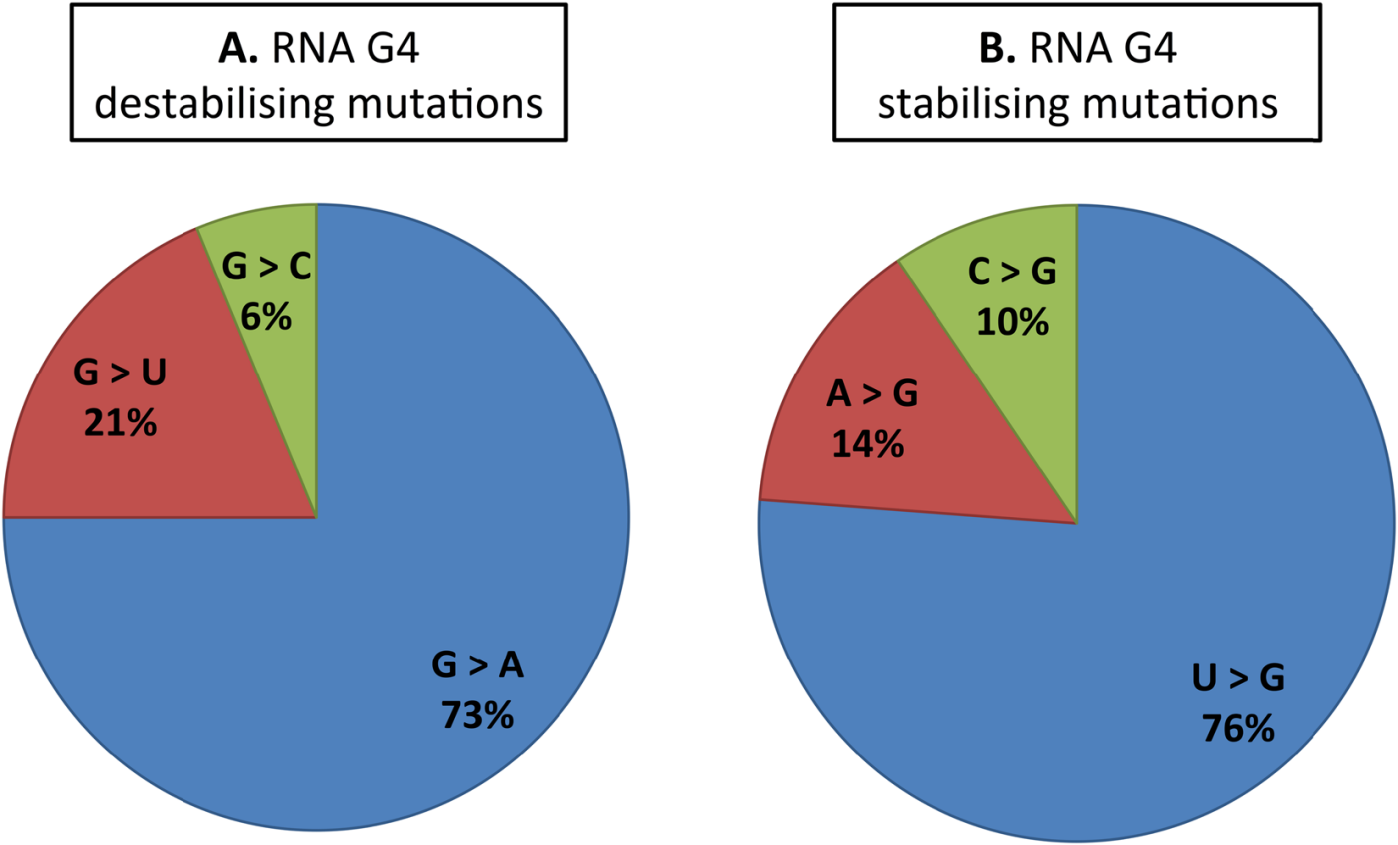
Pie chart demonstrating the frequency of different type of mutations in each of the RNA G4 (**A**) destabilising and (**B**) stabilising mutation groups.

We found 33 RNA G4 destabilising mutations among 217 overlaps between somatic mutations and predicted 5′ UTR G4s listed in the Supplementary Table 2. Based on the RNAfold predictions, these mutations remove one or more of the G-tetrads, increase the loop length or shift the RNA G4 position. A majority of them are transitions from guanine to adenine (Fig. 2A). On the other hand, 21 of the overlaps were found to be RNA G4 stabilising mutations (Supplementary Table 3). As predicted by RNAfold, in general, these mutations either increase the number of G-tetrads or shorten a loop length. Most of them are transversions from uracil to guanine (Fig. 2B). To evaluate the accuracy of the *in silico* results, one stabilising mutation in the 5′ UTR of *TAOK2* (TAO Kinase 2), one destabilising mutation in the 5′ UTR of *BCL2* (B-cell lymphoma 2) and two adjacent destabilising mutations in the 5′ UTR of *CXCL14* (C-X-C motif ligand 14) were selected for experimental validation.

### A point mutation destabilises the RNA G4 in the 5′ UTR of *BCL2*

**Figure 3:**
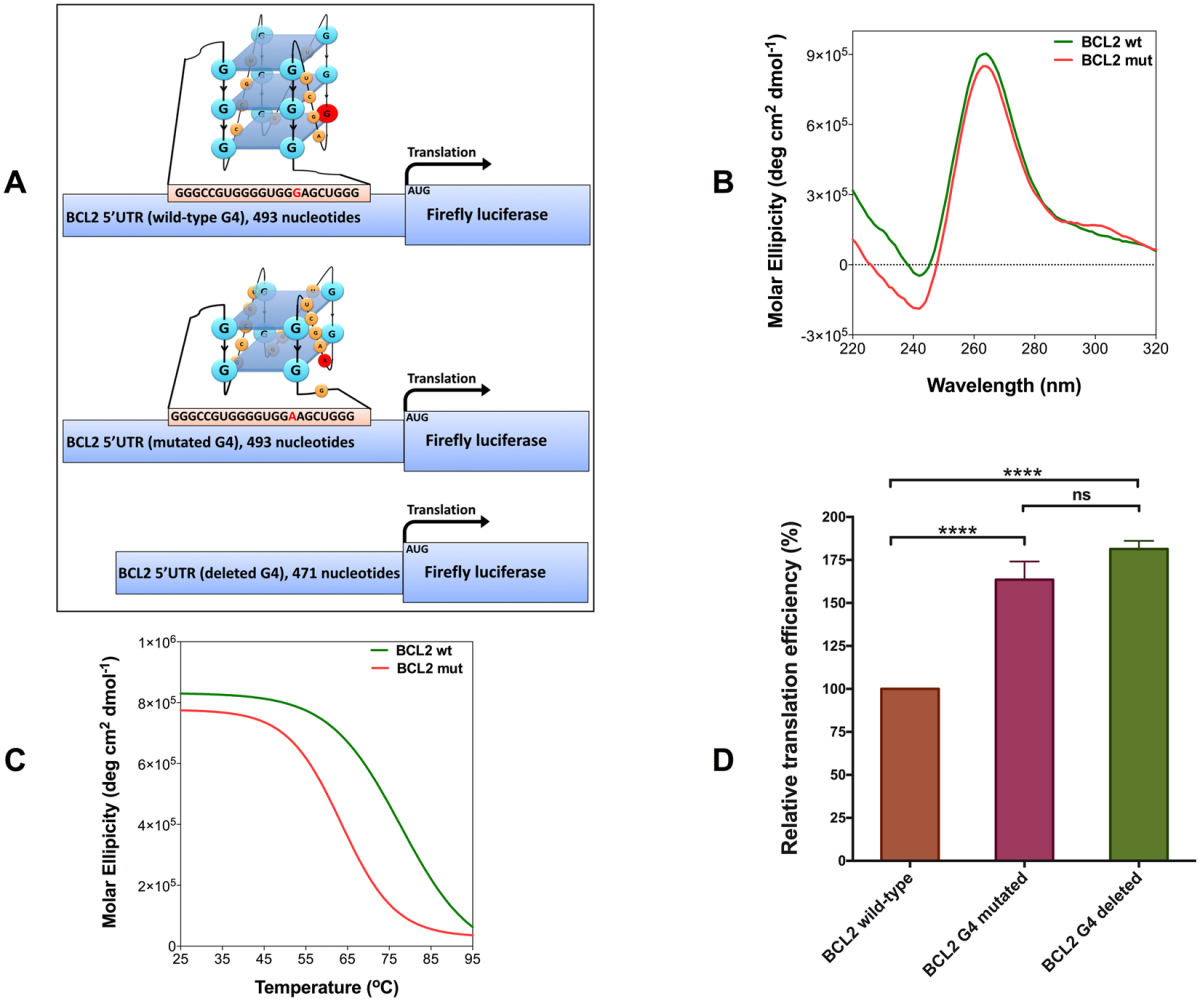
A point mutation in the 5′ UTR of *BCL2* destabilises the RNA G4 and affects its regulatory function. (**A**) Schematic representation of three different mRNAs of firefly luciferase reporter constructs. Wild-type construct contains full-length 5′ UTR of *BCL2*. In the G4-mutated construct, one of the guanines is replaced by adenine. To make the G4-deleted construct, 22 nucleotides that form the G4 were deleted. Schematic drawing of wild-type and mutated G4s are proposed based on RNAfold and QGRS-mapper predictions and *in vitro* results. (**B**) CD spectra of *BCL2* wild-type and mutated RNA G4 oligos in KCl. (**C**) CD melting curves of *BCL2* wild-type and mutated RNA G4 oligos at 260 nm. (**D**) Relative *in vitro* translation efficiency of the wild-type, G4-mutated and G4-deleted constructs as judged by measuring firefly luciferase enzymatic activity. Data were analysed using one-way-ANOVA (n ≥ 3, error bars represent the SEM, asterisks (*) indicate significance calculated by the Tukey multiple comparisons test, ns indicates non-significant).

It has been well demonstrated that the over-expression of *BCL2* proto-oncogene contributes to the resistance of cancer cells to apoptosis^27^. Our bioinformatics analysis identified a guanine to adenine point mutation in a patient with malignant lymphoma, which was predicted to destabilise the RNA G4 in the 5′ UTR of *BCL2* (Supplementary Table 2). It has been previously shown that the *BCL2* RNA G4 is very stable near physiological ionic and pH conditions and acts as a translational repressor in the 5′ UTR of the *BCL2* proto-oncogene^28^. To assess to what extent the mutation can destabilise the *BCL2* RNA G4, *in silico*, *in vitro* and *in cellulo* evaluations were conducted. RNAfold and QGRS-Mapper^14^ both predict the same RNA G4 with three G-tetrads for the wild-type sequence. However, RNAfold does not predict any G4 structure for the mutated *BCL2* G4 sequence, whereas QGRS-Mapper predicts a G4 structure with two G-tetrads (Fig. 3A; Supplementary Table 4).

CD spectroscopy is a well-established method to characterise the structure of G4s. A negative peak near 240 nm and a positive peak near 260 nm in the CD spectrum are characteristics of a parallel G4^29^. CD spectra of wild-type and mutated *BCL2* RNA G4 oligos were recorded in near physiological pH and potassium concentration. Both wild-type and mutant oligos can fold into a parallel G4 conformation (Fig. 3B). It is noteworthy that the CD spectrum of the mutated RNA G4 oligo is in accordance with the QGRS-Mapper result, which predicts an RNA G4 containing two G-tetrads for this oligo. CD melting at 260 nm was performed to compare the stability of wild-type and mutated *BCL2* RNA G4 oligos (Fig. 3C). Compared to the wild-type RNA G4 oligo, a 14 **°**C decrease in the *T*_m_ value of mutated oligo was observed (Table 1).

**Table 1:** Melting temperatures of RNA G4 oligos annealed in 100 mM KCl, 20 mM Tris-HCl, pH 7.5.

RNA G4 oligos		*T*_m_ (°C)^a^
*BCL2*	wt	78 ± 2.1
	mut	64 ± 0.2
Long *BCL2* ^b^	wt	74.5 ± 3.5
	mut	58 ± 2
*CXCL14*	wt	>90^c^
	mut	73 ± 0.5
*TAOK2*	wt	68 ± 0.5
	mut	75 ± 1

^a^ *T*_m_ values are expressed as mean ± SD (n ≥ 2).

^b^Long *BCL2* consists of 22 nucleotides predicted to form the RNA G4 and 20 extra nucleotides from each side.

^c^ *CXCL14* wt RNA G4 oligo could not be completely melted even at 95 °C.

Luciferase reporter assays were performed to evaluate the impact of the identified *BCL2* point mutation on this RNA G4’s function *in vitro*. Three constructs were generated by cloning full-length wild-type, G4-mutant and G4-deleted 5′ UTRs of *BCL2* immediately downstream of a T7 promoter and upstream of the firefly luciferase gene (Fig. 3A). For each construct, 5′-capped RNA transcripts were generated *in vitro* using T7 RNA polymerase. Equal transcript amounts from each construct were subjected to *in vitro* translation using nuclease-treated rabbit reticulocyte lysate and translation efficiency was determined by measuring firefly luciferase catalytic activity. The deletion of RNA G4 sequence from the 5′ UTR resulted in the highest relative translation efficiency (Fig. 3D), which supports the notion that the RNA G4 in the 5′ UTR of *BCL2* act as a translational repressor. Compared to the wild-type, a guanine to adenine point mutation in the 5′ UTR of *BCL2* resulted in a significant increase in translation efficiency (P < 0.0001), which is consistent with destabilisation of the RNA G4 structure.

**Figure 4:**
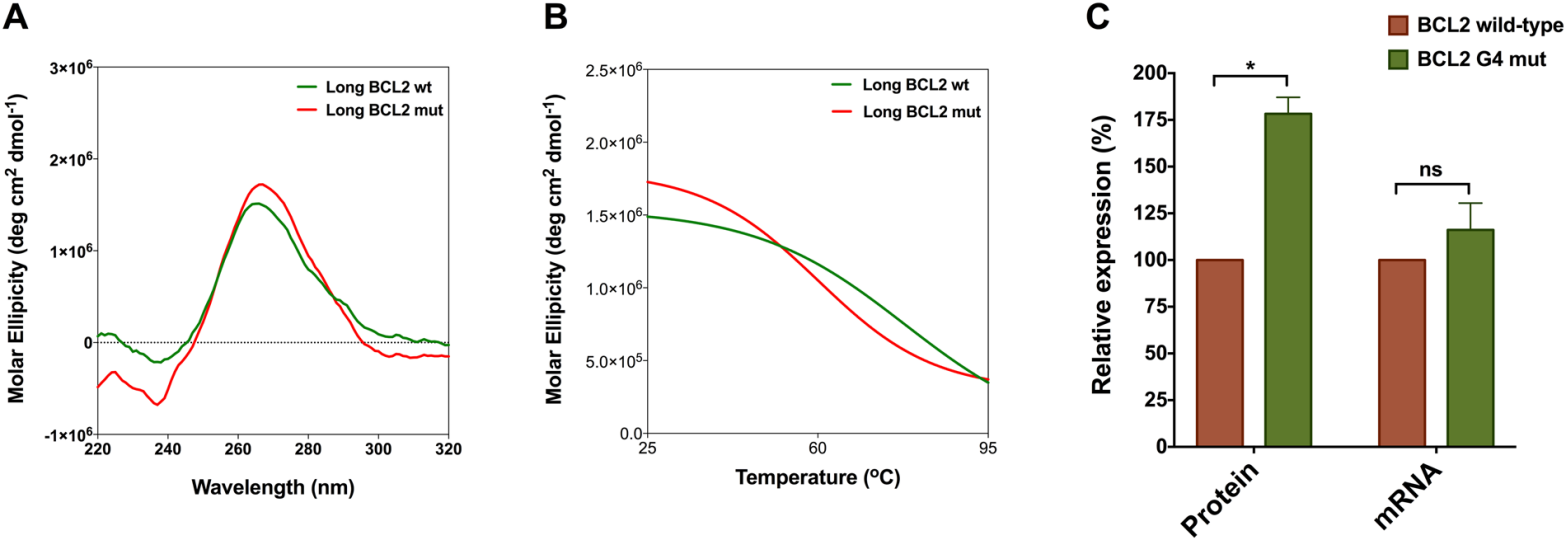
Further characterization of the RNA G4 destabilising mutation in the 5′ UTR of *BCL2* (**A**) CD spectra of wild-type and mutated *BCL2* oligomer which consist of 22 nucleotides predicted to form the G4 and 20 flanking nucleotides in KCl. (**B**) CD melting curves of wild-type and mutated *BCL2* oligomer at 260 nm. (**C**) Gene expression level of wild-type and G4-mutated construct at the protein level as determined by dual luciferase assay and the mRNA level as determined by RT-qPCR. Data were analysed using multiple t-test (n ≥ 2, error bars represent the SEM, asterisks (*) indicate significance and ns indicates non-significant).

Although *in silico* analysis using RNAfold and *in vitro* luciferase reporter assay have been performed in the context of the entire 5′ UTR of *BCL2*, we repeated the biophysical studies using 62-mer oligos of the wild-type and mutated *BCL2* RNA G4 that had 20 extra nucleotides from each side (Supplementary Table 1) to investigate if the flanking sequences can affect the G4 structure and stability. As recorded for the 22-mer wild-type and mutated oligos, CD spectroscopy showed a negative peak near 240 nm and a positive peak near 260 nm for both 62-mer wild-type and mutated oligos (Fig. 4A). CD melting of 62-mer oligos showed that the *T*_m_ value of the mutated oligo is 16.5 **°**C less than the wild-type oligo (Fig. 4B and Table 1). These observations suggest that even in the presence of the flanking sequences, wild-type and mutated *BCL2* RNA G4 oligos fold into G4 structures, however, the mutated G4 is less stable.

To investigate the effect of the mutation on the translation level *in cellulo* and to validate the *in vitro* reporter assay results, we performed dual luciferase assay. For this purpose, the T7 promoter was replaced by CMV promoter in the wild-type and G4-mutant constructs, which have been described earlier, and each of these constructs co-transfected into MCF-7 cells together with pRL-TK as a normalizing vector and luciferase activity was measured. We observed ~75% higher translation level from G4-mutant construct (Fig. 4C) which is almost the same as *in vitro* translation results (Fig. 3D). In order to confirm that the observed difference in the gene expression level is due to the post-transcriptional regulation by the destabilised G4 in the 5′ UTR rather than the transcriptional regulation, we performed RT-qPCR on the total RNA extracted from the same samples used for the dual luciferase assay. As shown in Fig. 4C, there is no significant difference between the wild-type and G4-mutant constructs at the mRNA expression level which verifies post-transcriptional regulation is responsible for the difference observed in the protein expression level.

### Two adjacent mutations destabilise the RNA G4 in the 5′ UTR of *CXCL14*

**Figure 5:**
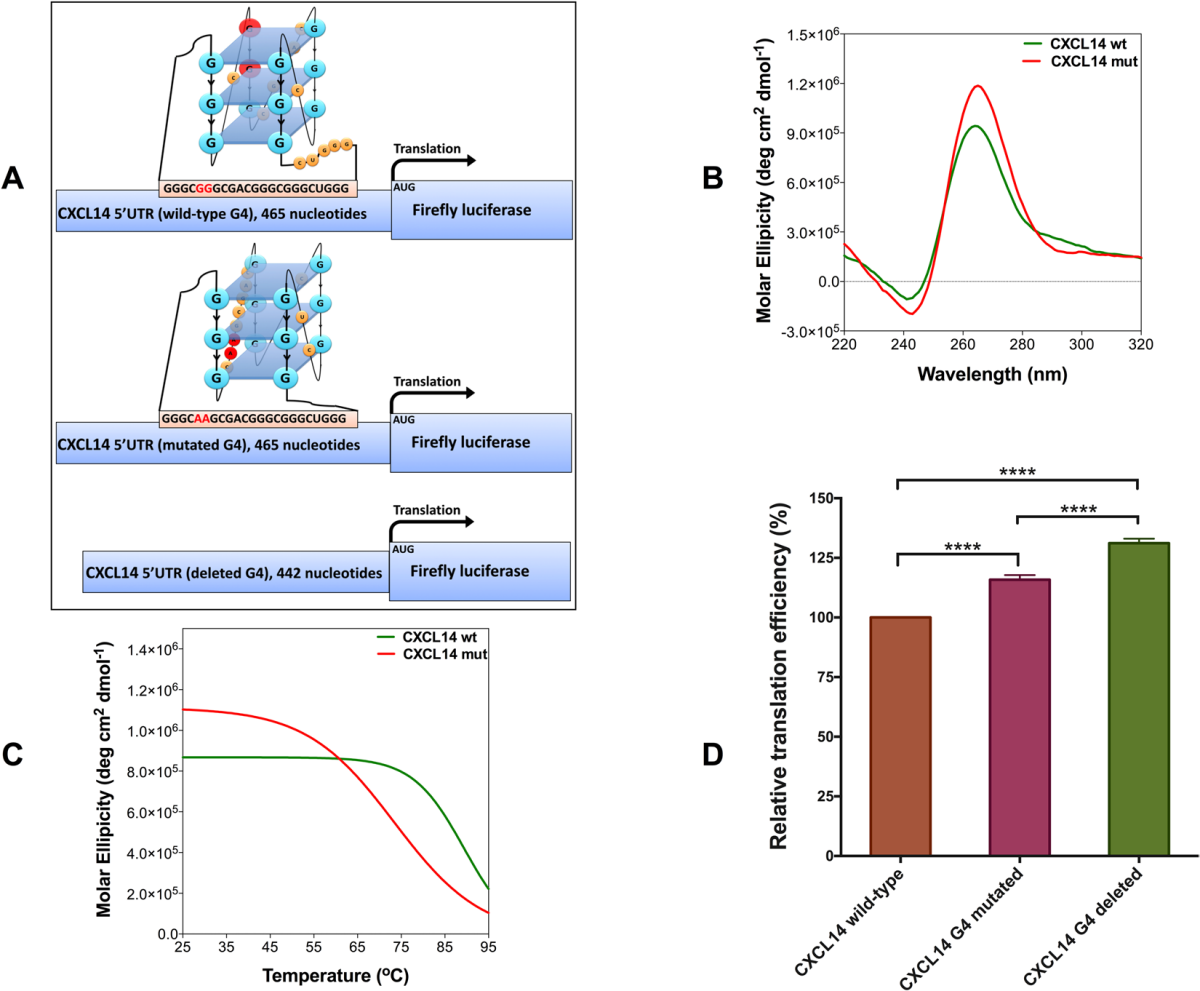
Two adjacent mutations in the 5′ UTR of *CXCL14* destabilise the RNA G4 and affect its regulatory function. (**A**) Schematic representation of three different mRNAs of firefly luciferase reporter constructs. Wild-type construct contains full-length 5′ UTR of *CXCL14*. In the G4-mutated construct, two guanines are replaced by two adenines. To make the G4-deleted construct, 23 nucleotides that predicted to form the RNA G4 were deleted. Schematic drawing of wild-type and mutated G4s are proposed based on RNAfold and QGRS-mapper predictions and *in vitro* results. (**B**) CD spectra of *CXCL14* wild-type and mutated RNA G4 oligos in KCl. (**C**) CD melting curves of *CXCL14* wild-type and mutated RNA G4 oligos at 260 nm. (**D**) Relative *in vitro* translation efficiency of the wild-type, G4-mutated and G4-deleted constructs as judged by measuring firefly luciferase enzymatic activity. Data were analysed using one-way-ANOVA (n ≥ 3, error bars represent the SEM, asterisks (*) indicate significance calculated by the Tukey multiple comparisons test).

The length of loops within an RNA G4 are inversely proportional to its stability^30^. We identified several mutations predicted to destabilise RNA G4s by lengthening one of the loops (Supplementary Table 2). These mutations are located in a G-rich region, which more than four G-runs exist. Theoretically, just four of the G-runs with shorter loops contribute to the RNA G4 structure. When a destabilising mutation disrupts one of the G-runs involved in the G4 structure, the fifth G-run, which is a few nucleotides away from the destabilised G4, contributes to the G4 formation and one of the loops lengthens. As an example, two adjacent guanine to adenine mutations in a patient with melanoma can disrupt one of the five G-runs in the 5′ UTR of *CXCL14* and destabilise the predicted RNA G4. *CXCL14* is a chemokine, which mainly contributes to the regulation of immune cell migration^31^. RNAfold predicts a total disruption of the RNA G4 caused by these mutations in the context of a full-length 5′ UTR. However, RNALfold, which scans for locally stable secondary structures, and QGRS-Mapper both predict an RNA G4 with a longer loop and less stability (Fig. 5A; Supplementary Table 4).

To investigate whether predicted wild-type and mutated *CXCL14* RNA G4s could fold into G4 structures *in vitro*, CD spectra of these oligos were recorded in near physiological pH and potassium concentration. The wild-type oligo was designed to consist of five G-runs, whereas in the G4-mutated oligo, two of the guanines were replaced by adenines in the second G-run (Fig. 5A; Supplementary Tables 1 and 4). The negative peak near 240 nm and the positive peak near 260 nm in the CD spectra of wild-type and G4-mutated oligos indicate that both oligos form parallel G4 structures (Fig. 5B). As the *CXCL14* RNA G4 has not been characterized *in vitro* before, to exclude the possibility of a false positive G4 signature, the CD spectroscopy was repeated while the potassium in the buffer was replaced by lithium. It has been shown previously that lithium does not support G4 structure formation as strongly as potassium. As shown in Supplementary Figure 1, a significant difference is observed between the CD spectrum of wild-type *CXCL14* RNA G4 in the presence of 100 mM of lithium compared with the same G4 in 100 mM of potassium. While CD spectroscopy results indicate both wild-type and mutated oligos can fold into parallel G4 structures, CD melting curves and *T*_m_ values of these two oligos show that the mutated oligo is less stable than the wild-type (Fig. 5C; Table 1). This might be because the mutations change the arrangement of G-runs and lengthen one of the loops, as predicted by RNALfold and QGRS-mapper (Fig. 5A; Supplementary Table 4).

To investigate the effect of these two mutations on the function of the *CXCL14* RNA G4 *in vitro*, three different luciferase reporter constructs were generated (Fig. 5A) as described earlier for *BCL2*. Quantitation of luciferase catalytic activity showed that the translational efficiency for the G4-mutated construct is higher than the wild-type construct and lower than the G4-deleted construct (P < 0.0001), which supports an effect of the destabilised RNA G4 in de-repressing translation (Fig. 5D).

### A point mutation stabilises the RNA G4 in the 5′ UTR of *TAOK2*

**Figure 6:**
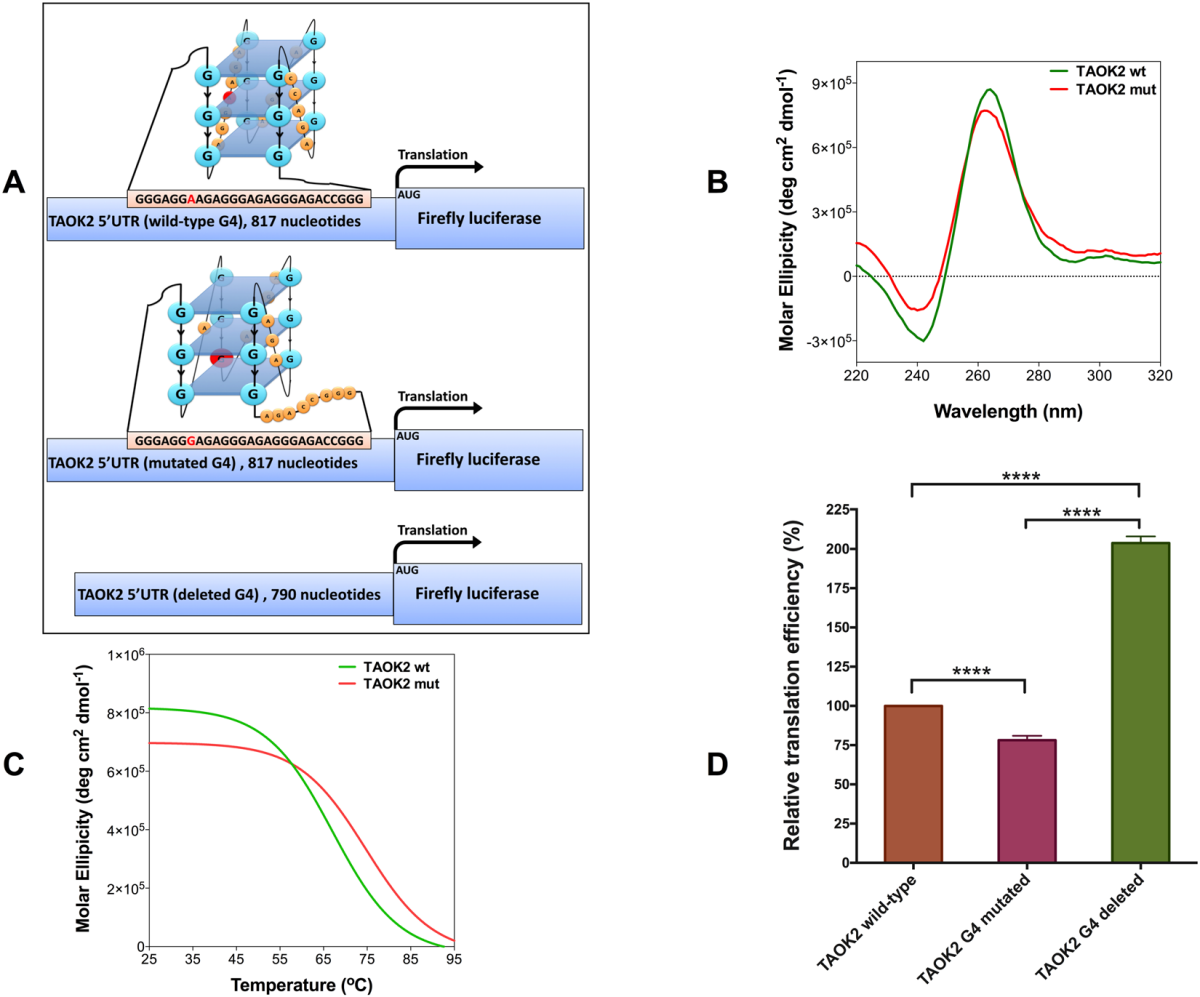
A point mutation in the 5′ UTR of *TAOK2* stabilises the RNA G4 and affect its regulatory function. (**A**) Schematic representation of three different mRNAs of firefly luciferase reporter constructs. Wild-type construct contains full-length 5′ UTR of *TAOK2*. In the G4-mutated construct, an adenine is replaced by a guanine. To make the G4-deleted construct, 27 nucleotides that predicted to form the G4 were deleted. Schematic drawing of wild-type and mutated G4s are proposed based on RNAfold and QGRS-mapper predictions and *in vitro* results. (**B**) CD spectra of *TAOK2* wild-type and mutated RNA G4 oligos in KCl. (**C**) CD melting curves of *TAOK2* wild-type and mutated RNA G4 oligos at 260 nm. (**D**) Relative *in vitro* translation efficiency of the wild-type, G4-mutated and G4-deleted constructs as judged by measuring firefly luciferase enzymatic activity. Data were analysed using one-way-ANOVA (n ≥ 3, error bars represent the SEM, asterisks (*) indicate significance calculated by the Tukey multiple comparisons test).

In addition to the destabilising mutations, our bioinformatics analyses predicted several mutations in the G-rich regions of 5′ UTRs that can stabilise potential RNA G4 structures (Supplementary Table 3). One of these mutations, which has been reported in a patient with colon cancer, overlaps a predicted RNA G4 in the 5′ UTR of *TAOK2*. This gene encodes a protein kinase, which involves in cell signalling pathways^32^ and the induction of apoptosis^33^. The mutation is an adenine to guanine located next to two adjacent guanines in the first loop. The mutation adds a G-run to the existing four G-runs and based on RNAfold and QGRS-Mapper predictions, it stabilises the RNA G4 by changing the arrangement of G-runs involved in the G4 structure formation and shortening one of the loops (Fig. 6A; Supplementary Table 4).

CD spectra of wild-type and G4-mutated *TAOK2* oligos showed that they can fold into a parallel G4 conformations in near physiological pH and potassium concentration (Fig. 6B), whereas in lithium-containing buffer, the G4 structure signature is much weaker (Supplementary Figure 1). CD melting curves of these oligos (Fig. 6C) showed that the *T*_m_ value of the G4-mutated *TAOK2* oligo is 7 °C higher than that of the wild-type oligo (Table 1), which further supports the increased stability of the RNA G4.

Three different luciferase reporter constructs were generated (Fig. 6A) as described earlier for *BCL2* and *CXCL14*, to evaluate the G4 stabilising effect of this mutation *in vitro*. Compared to the wild-type G4 construct, the G4-mutated construct showed 25% lower translation efficiency (P < 0.0001), whereas the G4-deleted construct showed a significant increase in translation efficiency (P < 0.0001) (Fig. 6D). These results indicate that the mutated RNA G4 is more efficient in acting as a translational suppressor motif, which could be the consequence of its elevated thermodynamic stability.

## Discussion

Cancer is considered as a multifactorial disease, where a combination of genetic and environmental factors contributes to cancer development, rather than a single gene mutation^3^. Most studies carried out to date have focused on the sequencing and characterization of mutations in protein-coding regions of the genome. However, knowledge regarding the effect of noncoding mutations in cancer development is very limited. Given that important regulatory elements, such as promoters and UTRs of mRNAs, are located in noncoding regions, it is likely that cancer driver mutations are found in these regions. Mutations in these regions can affect the structure and stability of regulatory motifs, including G4 structures. A number of G4s have been characterised as regulatory motifs in the promoters of many genes including proto-oncogenes, such as *c*-*KIT* ^34^ and *KRAS* ^35^. In addition to the promoters, there is evidence for regulatory functions of G4s in the 5′ UTR of several genes including *BCL2* ^28^ and *NRAS* ^21^ proto-oncogenes. Furthermore, a significant association between single nucleotide polymorphisms located in predicted G4 sequences and expression of the corresponding gene in individuals was previously demonstrated in a genome-wide study^36^. Here, for the first time, to the best of our knowledge, we demonstrate that noncoding mutations in cancer alter RNA G4s stability and affect their regulatory functions in the 5′ UTRs.

Using publically available whole-genome sequencing data, we found 217 mutations that overlap the potential G4-forming sequences in 5′ UTRs. To distinguish mutations that potentially alter G4 stability from inconsequential mutations, we evaluated the effects of the mutations on predicted G4 structures using RNAfold. This software considers the most important thermodynamic parameters, which contribute to RNA G4 stability^26^. To mimic the biological condition as much as possible, we examined the effect of each overlapping mutation in the context of the full-length 5′ UTR. We found 33 RNA G4 destabilising and 21 stabilising mutations and selected one stabilising mutation in the 5′ UTR of *TAOK2*, one destabilising mutation in the 5′ UTR of *BCL2* and two adjacent destabilising mutations in the 5′ UTR of *CXCL14* to validate experimentally. It is noteworthy that if the RNAfold was used initially to find the overlapping mutations instead of the Quadparser algorithm, we might have overlooked the stabilising mutations.

*BCL2* is a human proto-oncogene and belongs to the Bcl-2-family of proteins. These proteins are responsible for the physiological regulation of apoptosis, which is essential for development and tissue homeostasis^27^. Many examples exist where the expression of *BCL2* is elevated in human malignancies, particularly lymphomas. Several mechanisms contribute to the overexpression of *BCL2*, including chromosomal translocations and loss of endogenous microRNAs that repress *BCL2* expression^37^. In addition, it has been demonstrated that *cis*-regulatory mutations contribute to the dysregulation of the *BCL2* expression^38^. Balasubramanian *et al*. characterised a very stable RNA G4 in the 5′ UTR of *BCL2*, which was found to negatively regulate translation of the gene *in vitro* ^28^. Here, we report a mutation from a patient with malignant lymphoma that overlaps this RNA G4. Comparison between CD melting curves and *T*_m_ values of the wild-type and mutated *BCL2* RNA G4 show that the mutation destabilises this RNA G4 (Fig. 3C; Table 1). In order to rule out the possibility that the G4 flanking sequence might interfere with its formation and stability, we included 20 adjacent nucleotides from each side and repeated the biophysical study using 62-mer wild-type and mutated oligos. As shown in Fig. 4A, the CD spectra of both 62-mer wild-type and mutated oligos have the characteristic peaks for parallel propeller G4 structure. In addition, we observed that the difference between *T*_m_ values of the 62-mer wild-type and mutated oligos are almost the same as the 22-mer oligos (Table 1) denoting that the flanking sequence of *BCL2* RNA G4 does not interfere with this G4 structure and stability. Using *in vitro* luciferase reporter assays, we show that this mutation significantly elevates the translation efficiency (Fig. 3D). We verified this result by performing dual luciferase assay in MCF-7 cell line. We observed ~75% increase in the gene expression at the protein level for the *BCL2* G4-mutant construct, whereas there is not a significant difference in the gene expression at the mRNA level between the *BCL2* G4-mutant and wild-type constructs. Taken together, these results suggest the guanine to adenine point mutation in one of the G-tetrads of *BCL2* RNA G4 does not completely disrupt the G4 structure, but significantly decreases its stability and affects its regulatory capability as a translational repressor in the 5′ UTR of the *BCL2* proto-oncogene.

We also experimentally validated the effect of two adjacent guanine to adenine destabilising mutations in a patient with melanoma that overlap a predicted RNA G4 in the 5′ UTR of *CXCL14*. This gene belongs to the CXC subfamily of chemokines, which are involved in immunoregulatory and inflammatory processes. Tumor suppressing and promoting activities have been reported for *CXCL14*, depending on the type and the stage of cancer^39, 40^. We showed that the mutations decrease the *T*_m_ value of the mutated RNA G4 oligo (Table 1) and increase the translation efficiency of the gene (Fig. 5D) probably because they rearrange the G4 structure by lengthening one of the loops and making the G4 structure less stable.

Contrary to mutations that destabilise G4 structures, some mutations in G-rich sequences can stabilise G4 structures and in turn affect their physiological function. Among 21 stabilising mutations listed in the Supplementary Table 3, we experimentally validated the effect of a mutation on a predicted G4 in the 5′ UTR of *TAOK2*. This gene is one of the TAO (thousand-and-one amino acids) protein kinases that involves in several different cellular processes including cell-signalling pathways^32, 41, 42^. TOAK2 has been suggested to be involved in the induction of apoptosis^33^. Here, we demonstrated that the predicted G4 sequence in the 5′ UTR of *TAOK2* folds into a parallel quadruplex *in vitro* (Fig. 6B) and a mutation in a patient with colon cancer increases the *T*_m_ value of the mutated *TAOK2* RNA G4 oligo (Table 1) and decreases the translation efficiency compared to the wild-type (Fig. 6D). The elevated stability in the mutated *TAOK2* RNA G4 might be attributed to the reorganisation of G-runs caused by the mutation. There are two successive guanines in the first loop of *TAOK2* RNA G4. The adenine to guanine mutation next to these guanines together form a new G-run that shifts the last G-run outside the RNA G4. Therefore, the first loop is shortened and the RNA G4 becomes more stable.

The results presented in this paper are based on bioinformatics analyses and *in vitro* experiments that show somatic mutations in cancer patients can alter RNA G4 structure stability in the 5′ UTR of mRNAs and affect its regulatory function. This study provides evidence for the concept that these mutations might contribute to cancer development. Recently, Balasubramanian and colleagues developed a method for high-throughput sequencing of DNA G4s and identified more than 700,000 unique G4 structures in the genome, which is more than twice the number of G4s predicted by computational methods^43^. This dataset represents a good starting point for a deeper analysis of the effect of mutations on G4 structure and function. Analysing larger datasets might obviate the lack of recurrency among the G4 stabilising or destabilising mutations observed in this study. Finally, considering that G4 binding ligands can stabilise G4 structures, it will be important to investigate whether they can restore the stability of a destabilised G4. Such ligands may include small organic molecules or alternately protein ligands such as antibody fragments which can be generated by *in vitro* selection technology^23, 44^. Further understanding of the relationship of the pathogenicity of variants within G4 structures will improve the diagnostic potential of genome sequencing for both somatic and germline mutations and may form a basis for new therapeutic targets.

## Material and Methods

The sequences of all of the oligonucleotides used in the biophysical studies, primers and 5′ UTRs are provided in Supplementary Table 1.

### Bioinformatics

Somatic mutation data from whole cancer genomic sequences were obtained from three publicly available data sources: TCGA, ICGC and Alexandrov *et al*.^25^. The single base substitution data from the ICGC were obtained from the ICGC data portal (release 16) and the FTP site provided by Alexandrov *et al*. These mutations were used directly for the analysis. For samples obtained from TCGA, mutations were called from BAM files obtained from CGHub using Strelka with default parameters^45^. The genomic coordinates of somatic variants are reports using NCBI build 37, aka hg19.

Quadparser algorithm^13^ was used to identify potential G4-forming sequences in the NCBI build 37 version of the human genome sequence. UCSC Table Browser^46^ was used to find the overlaps between identified mutations and potential G4-forming sequences in the 5′ UTR regions. The effect of a mutation on the thermodynamic stability of an RNA G4 was evaluated using RNAfold and RNALfold^26^ version 2.1.9 in the context of the full-length 5′ UTR while GU base paring was not allowed. In addition to RNAfold, the QGRS-Mapper^14^ online tool was used to investigate the effect of mutations on the RNA G4-forming sequences in the 5′ UTR of *BCL2*, *CXCL14* and *TAOK2*.

### Circular dichroism spectroscopy

Circular dichroism spectra were recorded as previously described^47^. Briefly, RNA oligos were annealed by heating at 90 °C for 10 minutes and slowly cooling down to room temperature. CD spectra were recorded at 25 **°**C on an Aviv 215S circular dichroism spectrometer equipped with a Peltier temperature controller. RNA G4 samples of the desired sequence were prepared at 5 µM in the G4 folding buffer (100 mM KCl, 20 mM Tris HCl pH 7.5). KCl was replaced by LiCl in the control samples. Four scans were accumulated over the wavelength range 220–320 nm in a 0.1 cm path length cell at the standard sensitivity, data pitch 0.1 nm, continuous scanning mode, scanning speed 100 nm min^−1^, response 4 sec, and bandwidth 1 nm. Buffers alone were scanned and these spectra subtracted from the average scans for each sample. *T*_m_ analysis was recorded by CD at 260 nm and heated at 1 **°**C min^−1^ over the temperature range 25–95 **°**C. CD and melt curve spectra were collected in units of millidegrees, normalized to the total species concentrations and expressed as molar ellipticity units (deg × cm^2^ dmol^−1^). Each CD spectrum was smoothed by averaging ten neighbour points using Prism 6. Sigmoidal dose-response curves (variable slope) were fitted to the melt curve data using Prism 6.

### Construction of plasmids

Overlap extension PCR was used to clone either T7 or CMV promoter upstream of multiple restriction sites of pGL3 basic plasmid (Promega). The sequences of the 5′ UTRs of *BCL2*, *CXCL14* and *TAOK2* were obtained from the UCSC Genome Browser^48^ based on the NCBI build 37 version of the human genome sequence. The synthetically synthesised full-length 5′ UTRs of each candidate was purchased from GenScript and inserted in the KpnI and HindIII restriction sites of T7 or CMV pGL3 plasmid. These constructs are considered as wild-types. G4-mutated and G4-deleted constructs were created based on the wild-type constructs for each 5′ UTR. The NEBaseChanger^TM^ online tool provided by New England BioLabs was used to design primers for making desired point mutations in the G4-forming sequences and also deleting the entire G4-forming sequences from the 5′ UTRs.

### *In vitro* transcription

A gene fragment containing T7 promoter and the full-length 5′ UTR attached to the firefly luciferase gene was provided from each of the wild-types, G4-mutated and G4-deleted constructs by PCR amplification. The size of each fragment was examined on a 1% agarose gel followed by gel purification. An additional step of PCR clean up was performed and RNase inhibitor (Ambion) at a final concentration of 1 U/μL was added to each sample. The PCR products were used as templates to produce 5′-capped transcripts *in vitro* using mMESSAGE mMACHINE T7 kit (Ambion). Transcripts were DNase treated and purified using the RNeasy Mini Kit (QIAGEN). The size and integrity of the transcripts were confirmed on a 1% agarose gel. The concentration of each set of transcripts was determined by NanoDrop.

### *In vitro* translation and luciferase assay

Nuclease-treated rabbit reticulocyte lysate (Promega) was used to perform the cell-free translation. 150 ng/μL (final concentration) of the *in vitro* transcribed 5′-capped mRNA containing the full-length 5′ UTR attached to the firefly luciferase coding sequence was translated according to the manufacturer’s protocol. In order to measure the firefly luciferase activity, 5 μL of the translation reaction was added to 75 μL of luciferase assay reagent (Promega) at room temperature. The reaction was quickly mixed and luminescence was measured using CLARIO star microplate reader (BMG LABTECH).

### Dual luciferase assay and RT-PCR

MCF-7 cells were seeded in 12-well plates (Corning) and incubated until they reached to 70–80% confluency. The cells were co-transfected with pRL-TK (Promega) normalizing vector, which expresses renilla, and the CMV pGL3 plasmids constructs, which prepared as described earlier and express firefly, with the ratio of 1 to 7 using Lipofectamine 3000 (Thermo Fisher Scientific) according to the manufacturer’s protocol. When cells reached to 90–95% confluency, renilla and firefly luciferase activities were measured using Dual-Luciferase Reporter Assay System (Promega) following the manufacturer’s protocol in the CLARIO star microplate reader (BMG LABTECH). Total RNA was extracted from the remaining cells using RNeasy mini kit (QIAGEN). 200 ng of each total RNA sample was reverse-transcribed using SuperScript III First-Strand Synthesis System (Thermo Fisher Scientific). The cDNA from each sample was used to perform RT-qPCR using LightCycler 480 SYBR Green I Master reagents (Roche) and a LightCycler 480 SYBR device (Roche). Primer sets are listed in the Supplementary Table 1. The relative mRNA expression level was calculated using the following mathematical model^49^: Ratio = (*E*_target_)^ΔCP^_target_^(control-sample)^/(*E*_reference_)^ΔCP^_reference_^(control-sample)^ where firefly and renilla were considered as the target and reference genes, respectively. GAPDH expression in each sample was considered as the control.

## Electronic supplementary material

Supplementary Dataset 1

Supplementary Information

## References

[1] Cancer Genome Atlas Research, N (2013). The Cancer Genome Atlas Pan-Cancer analysis project. Nat Genet.

[2] International Cancer Genome, C (2010). International network of cancer genome projects. Nature.

[3] Stratton MR, Campbell PJ, Futreal PA (2009). The cancer genome. Nature.

[4] Druker BJ (2008). Translation of the Philadelphia chromosome into therapy for CML. Blood.

[5] Little PF (2005). Structure and function of the human genome. Genome Res.

[6] Weinhold N, Jacobsen A, Schultz N, Sander C, Lee W (2014). Genome-wide analysis of noncoding regulatory mutations in cancer. Nat Genet.

[7] Puente XS (2015). Non-coding recurrent mutations in chronic lymphocytic leukaemia. Nature.

[8] Fredriksson NJ, Ny L, Nilsson JA, Larsson E (2014). Systematic analysis of noncoding somatic mutations and gene expression alterations across 14 tumor types. Nat Genet.

[9] Bochman ML, Paeschke K, Zakian VA (2012). DNA secondary structures: stability and function of G-quadruplex structures. Nat Rev Genet.

[10] Karsisiotis AI, O’Kane C, Webba da Silva M (2013). DNA quadruplex folding formalism–a tutorial on quadruplex topologies. Methods.

[11] Zhang DH (2010). Monomorphic RNA G-quadruplex and polymorphic DNA G-quadruplex structures responding to cellular environmental factors. Biochemistry.

[12] Todd AK, Johnston M, Neidle S (2005). Highly prevalent putative quadruplex sequence motifs in human DNA. Nucleic Acids Res.

[13] Huppert JL, Balasubramanian S (2005). Prevalence of quadruplexes in the human genome. Nucleic Acids Res.

[14] Kikin O, D’Antonio L, Bagga PS (2006). QGRS Mapper: a web-based server for predicting G-quadruplexes in nucleotide sequences. Nucleic Acids Res.

[15] Maizels N, Gray LT (2013). The G4 genome. PLoS Genet.

[16] Agarwala P, Pandey S, Mapa K, Maiti S (2013). The G-quadruplex augments translation in the 5′ untranslated region of transforming growth factor beta2. Biochemistry.

[17] Bugaut A, Balasubramanian S (2012). 5′-UTR RNA G-quadruplexes: translation regulation and targeting. Nucleic Acids Res.

[18] Beaudoin JD, Perreault JP (2010). 5′-UTR G-quadruplex structures acting as translational repressors. Nucleic Acids Res.

[19] Arora A (2008). Inhibition of translation in living eukaryotic cells by an RNA G-quadruplex motif. RNA.

[20] Morris MJ, Basu S (2009). An unusually stable G-quadruplex within the 5′-UTR of the MT3 matrix metalloproteinase mRNA represses translation in eukaryotic cells. Biochemistry.

[21] Kumari S, Bugaut A, Huppert JL, Balasubramanian S (2007). An RNA G-quadruplex in the 5′ UTR of the NRAS proto-oncogene modulates translation. Nat Chem Biol.

[22] Biffi G, Tannahill D, McCafferty J, Balasubramanian S (2013). Quantitative visualization of DNA G-quadruplex structures in human cells. Nat Chem.

[23] Biffi G, Di Antonio M, Tannahill D, Balasubramanian S (2014). Visualization and selective chemical targeting of RNA G-quadruplex structures in the cytoplasm of human cells. Nat Chem.

[24] Biffi G, Tannahill D, Miller J, Howat WJ, Balasubramanian S (2014). Elevated levels of G-quadruplex formation in human stomach and liver cancer tissues. PLoS One.

[25] Alexandrov LB (2013). Signatures of mutational processes in human cancer. Nature.

[26] Lorenz R (2013). 2D meets 4G: G-quadruplexes in RNA secondary structure prediction. IEEE/ACM Trans Comput Biol Bioinform.

[27] Cory S, Huang DC, Adams JM (2003). The Bcl-2 family: roles in cell survival and oncogenesis. Oncogene.

[28] Shahid R, Bugaut A, Balasubramanian S (2010). The BCL-2 5′ untranslated region contains an RNA G-quadruplex-forming motif that modulates protein expression. Biochemistry.

[29] Paramasivan S, Rujan I, Bolton PH (2007). Circular dichroism of quadruplex DNAs: applications to structure, cation effects and ligand binding. Methods.

[30] Halder K, Wieland M, Hartig JS (2009). Predictable suppression of gene expression by 5′-UTR-based RNA quadruplexes. Nucleic Acids Res.

[31] Lu J, Chatterjee M, Schmid H, Beck S, Gawaz M (2016). CXCL14 as an emerging immune and inflammatory modulator. J Inflamm (Lond).

[32] Chen Z, Cobb MH (2001). Regulation of stress-responsive mitogen-activated protein (MAP) kinase pathways by TAO2. J Biol Chem.

[33] Zihni C (2007). Prostate-derived sterile 20-like kinase 1-alpha induces apoptosis. JNK- and caspase-dependent nuclear localization is a requirement for membrane blebbing. J Biol Chem.

[34] Fernando H (2006). A conserved quadruplex motif located in a transcription activation site of the human c-kit oncogene. Biochemistry.

[35] Cogoi S, Xodo LE (2006). G-quadruplex formation within the promoter of the KRAS proto-oncogene and its effect on transcription. Nucleic Acids Res.

[36] Baral A (2012). Quadruplex-single nucleotide polymorphisms (Quad-SNP) influence gene expression difference among individuals. Nucleic Acids Res.

[37] Yip KW, Reed JC (2008). Bcl-2 family proteins and cancer. Oncogene.

[38] Mathelier A (2015). Cis-regulatory somatic mutations and gene-expression alteration in B-cell lymphomas. Genome Biol.

[39] Hara T, Tanegashima K (2012). Pleiotropic functions of the CXC-type chemokine CXCL14 in mammals. J Biochem.

[40] Benarafa C, Wolf M (2015). CXCL14: the Swiss army knife chemokine. Oncotarget.

[41] Chen Z, Hutchison M, Cobb MH (1999). Isolation of the protein kinase TAO2 and identification of its mitogen-activated protein kinase/extracellular signal-regulated kinase kinase binding domain. J Biol Chem.

[42] Coffey ET (2014). Nuclear and cytosolic JNK signalling in neurons. Nat Rev Neurosci.

[43] Chambers VS (2015). High-throughput sequencing of DNA G-quadruplex structures in the human genome. Nat Biotechnol.

[44] Lee CM, Iorno N, Sierro F, Christ D (2007). Selection of human antibody fragments by phage display. Nat Protoc.

[45] Saunders CT (2012). Strelka: accurate somatic small-variant calling from sequenced tumor-normal sample pairs. Bioinformatics.

[46] Karolchik D (2004). The UCSC Table Browser data retrieval tool. Nucleic Acids Res.

[47] Moye AL (2015). Telomeric G-quadruplexes are a substrate and site of localization for human telomerase. Nat Commun.

[48] Kent WJ (2002). The human genome browser at UCSC. Genome Res.

[49] Pfaffl MW (2001). A new mathematical model for relative quantification in real-time RT-PCR. Nucleic Acids Res.

