# Supplementary material for: Cancer-associated noncoding mutations affect RNA G-quadruplex-mediated regulation of gene expression
Source: Sci Rep. 2017 Apr 6;7:708. doi: 10.1038/s41598-017-00739-y (PMC5429658; doi:10.1038/s41598-017-00739-y)
Supplement: Supplementary file 2 — Supplementary Information [file 41598_2017_739_MOESM2_ESM.pdf]

Cancer-associated noncoding mutations affect RNA G-quadruplex-mediated regulation of gene expression

Mahdi Zeraati[1,2], Aaron L. Moye[3], Jason W. H. Wong[4], Dilmi Perera[4], Mark J. Cowley[1,2], Daniel U. Christ[1,2], Tracy M. Bryan[3], Marcel E. Dinger [1,2 *]

[1] Garvan Institute of Medical Research, Sydney, NSW, 2010, Australia

[2] St Vincent's Clinical School, Faculty of Medicine, University of New South Wales, Sydney, NSW, 2052, Australia

[3] Children's Medical Research Institute, University of Sydney, Sydney, NSW, 2145, Australia

[4] Prince of Wales Clinical School, Faculty of Medicine, University of New South Wales, Sydney, NSW, 2052, Australia

Supplementary Table 1. List of sequences

BCL2

Name	Sequence (5' → 3')
BCL2 5'UTR	UUUCUGUGAAGCAGAAGUCUGGGAAUCGAUCUGGAAAUCCUCCUAA UUUUUACUCCCUCUCCCCGCGACUCCUGAUUCAUUGGGAAGUUUC AAAUCAGCUAUAACUGGAGAGUGCUGAAGAUUGAUGGGAUCGUUG CCUUAUGCAUUUGUUUUGGUUUUACAAAAAGGAAACUUGACAGAGG AUCAUGCUGUACUUAAAAAAUACAACAUCACAGAGGAAGUAGACUG AUAUUAACAAUACUUACUAAUAAUAACGUGCCUCAUGAAAUAAAGAU CCGAAAGGAAUUGGAAUAAAAAUUUCCUGCAUCUCAUGCCAAGGGG GAAACACCAGAAUCAAGUGUUCCGCGUGAUUGAAGACACCCCCUCG UCCAAGAAUGCAAAGCACAUCCAAUAAAAUAGCUGGAUUAUAACUCC UCUUCUUUCUCUGGGGGCCGUGGGGUGGGAGCUGGGGCGAGAGG UGCCGUUGGCCCCCGUUGCUUUUCCUCUGGGAAGG
Fwd primer for deleting BCL2 G4	GCGAGAGGTGCCGTTGGC
Rev primer for deleting BCL2 G4	CCAGAGAAAGAAGAGGAGTTATAATCCAGC
Fwd primer for mutating BCL2 G4	CGTGGGGTGGaAGCTGGGGCG
Rev primer for mutating BCL2 G4	GCCCCCAGAGAAAGAAGAGGAGTTATAATCC
Wild type BCL2 RNA G4 for biophysical study	r(GGGCCGUGGGGUGGGAGCUGGG)
Mutated BCL2 RNA G4 for biophysical study	r(GGGCCGUGGGGUGGAAGCUGGG)
Wild type **long** BCL2 RNA G4 for biophysical study	r(AACUCCUCUUCUUUCUCUGGGGGCCGUGGGGUGGGAGCUGGGG CGAGAGGUGCCGUUGGCCC)
Mutated **long** BCL2 RNA G4 for biophysical study	r(AACUCCUCUUCUUUCUCUGGGGGCCGUGGGGUGGAAGCUGGGGC GAGAGGUGCCGUUGGCCC)
Fwd primer for the RT-qPCR of firefly	CTGAGTACTTCGAAATGTCCGTTCGG
Rev primer for the RT-qPCR of firefly	CTCCGATAAATAACGCGCCCAACACC
Fwd primer for the RT-qPCR of renilla	GAGCCAGTAGCGCGGTGTATTATACC
Rev primer for the RT-qPCR of renilla	CCAAACAAGCACCCCAATCATGGC
Fwd primer for the RT-qPCR of GAPDH	GCTGAACGGGAAGCTCAC
Rev primer for the RT-qPCR of GAPDH	CCTCCGACGCCTGCTTC

CXCL14

Name	Sequence (5' → 3')
CXCL14 5'UTR	AAUGUGUGCGCGCUGUGGUAUGGGUGUGCAAGUGUGCGAAGGCG<br>GCGUGUUGUGUGAGCGAGAGGGUAGCGGAUGUGUGUGUGCGUGU<br>GCGCGCGUGGCUCCGGGUGUGCGCCGCUGCGAUAGCGGGUCCUU<br>UCCCGGGGCGGGCGACGGGCGGGCUGGGAAGGUCUCCUCCCCUC<br>ACCACAUUGAGAAAUCUCAGUGAGUCACCGAGUGGUUCUGCAUAUU<br>AAUGAGCUCGCUCGCUGCGAGGGCAGGAGCGGAUUUAAAAGAGGC<br>CAGGGCGGGCGGAGGGAGGCUGUGGAGAGAGCGCGGAGACAAGC<br>GCAGAGCGCAGCGCACGGCCACAGACAGCCCUGGGCAUCCACCGA<br>CGGCGCAGCCGGAGCCAGCAGAGCCGGAAGGCGCGCCCCGGGCAG<br>AGAAAGCCGAGCAGAGCUGGGUGGCGUCUCCGGGCCGCCGCUCCG<br>ACGGGCCAGCGCCCUCCCC
Fwd primer for deleting CXCL14 G4	CGGGAAGGCCCCCCCCAC
Rev primer for deleting CXCL14 G4	CGGGGGCCCGCTTCGCGC
Fwd primer for mutating CXCL14 G4	CCCCCGGGGCaaGCGACGGGCGG
Rev primer for mutating CXCL14 G4	CCCGCTTCGCGCGGCGCC
Wild type CXCL14 RNA G4 for biophysical study	r(GGGCGGGCGACGGGCGGGCUGGG)
Mutated CXCL14 RNA G4for biophysical study	r(GGGCAAGCGACGGGCGGGCUGGG)

TAOK2

Name	Sequence (5' → 3')
TAOK2 5'UTR	UCUCCAUCUUGGAAUUGGGAGGAAGAGGGAGAGGGAGACCGGGAC<br>GAGACCGGGGCUGUGGUGCGGAGAGAGGCUGAGACGGAGAAGAG<br>GAGAGGCAGAGAGGGCGCGGGGACCGUCAGCAGCACCUUAGCUAC<br>AAUCGUUCAGCUAUUCUCGGAAGAGAGAAGGGAGAGGGAGGAGGC<br>CGGGGCGGGAGUGGGGGCUGUCACCCUCGGACCCCGGCGUGAGA<br>GGGGCCGUGCGGCCGGACGUCCUCGGGGUGGGCCCCCAGUCGGU<br>GGCCGAAGACCUACAGCUCAGGCCCCUGGGUCCCAAAUUUCCAGG<br>CUUUGCCCCUCCUCCUUUCUCAGAUACCCGGGUAACAGUCCUCAUA<br>GUCCAGAUAUCCGGGACUCGGGUCCCAACCUCUCUAAACCUGGGU<br>CUCUGUUUCAUAGAAUUUCAAAUAUCAGGUUCAGGCCCCUGCGUGC<br>ACCAGUAUCCGGGGUUCAUUCCCCGGGCGUUCAAAUAUCGGAUUC<br>AGUCUCCAUCCCGUUCAGAUAUUCGGGGUUCAGACCCCACAAUCAG<br>AAAUCCGGAAUUCGGCAGCUGUCGCCCUCGACGAGGGGGAGGACU<br>GGACCGCGAGGUCAGAUUAGGUUGUCACCCCCUCCCCUCCAGGGG<br>AGGCUUCCCGGGCCCGCCCCUCAGGAAGGGCGAAAGCCGAGGAAG<br>AGGUGGCAAGGGGAAAGGUCUCCUUGCCCCUCUCCCUGCUUGGCA<br>GAGCCGCUGGAGGACCCCAGGCGGAAGCGGAGGCGCUGGGGCAC<br>CAUAGUGACCCCUACCAGGCCAGGCCCCACUCUCAGGGCCCCCAG<br>GGGCCACC
Fwd primer for deleting TAOK2 G4	GACCGGGACGAGACCGGG
Rev primer for deleting TAOK2 G4	AATTCCAAGATGGAGAGGTGGCC
Fwd primer for mutating TAOK2 G4	AATTGGGAGGgAGAGGGAGAG
Rev primer for mutating TAOK2 G4	CCAAGATGGAGAGGTGGC
Wild type TAOK2 RNA G4 for biophysical study	r(GGGAGGAAGAGGGAGAGGGAGACCGGG)
Mutated TAOK2 RNA G4 for biophysical study	r(GGGAGGGAGAGGGAGAGGGAGACCGGG)

Construction of T7 pGL3 plasmid and making templates for the in vitro transcription

Name	Sequence (5' → 3')
Fwd primer for colony PCR (RVprim3)	CTAGCAAAATAGGCTGTCCC
Rev primer for colony PCR (GLprim2)	TGGAAGACGCCAAAAACATAAAG
Fwd primer for cloning T7 promoter upstream of multiple cloning site (SmaI-T7-col pGL3 fwd)	actcactataggGTACCGAGCTCTTACGCG
Rev primer for cloning T7 promoter upstream of multiple cloning site (SmaI-T7-col pGL3 rev)	cgtattacccgggTATCGATAGAGAAATGTTCTGGC
Fwd primer for PCR amplification of the 5'UTR plus luciferase fragments (pGL3 SmaI T7 fwd)	CGATACCCGGGTAATACGACTCACTATAGGG
Rev primer for PCR amplification of the 5'UTR plus luciferase fragments (pGL3 luc XbaI rev)	GACTCTAGAATTACACGGCGATCTTTCCG

Supplementary Table 2. 5' UTR G4 destabilising mutations

Mutation details				G4 sequence [a] (5' → 3')			ΔG (kcal/mol) [b]	
**Mutation Coordinate [c]**	**Patient's ID**	**Gene**	**Cancer**	**Strand**	**Wild-type**	**Mutant**	**Wild-type**	**Mutant**
C > T chr15:50647428	PD3989a	GABPB1	Breast	-	GGGCCGGGUCCGGGAGGG	GGGCCGGAUCCGGGAGGG	-161.27	-150.30
C > G chr17:73511632	PD7321a	CASKIN2	Breast	-	GGGAGGGAGGGGGGAGGG	GGGAGGCAGGGGGGAGGG	-237.77	-231.89
G > A chr17:40834722	DO45087	CNTNAP1	Liver	+	GGGAGGGAAGGGUGGG	GGGAGGGAAAGGUGGG	-76.99	-53.11
C > T chr8:28347804	DO23534	FBXO16	Liver	-	GGGAGGGGUGAGGGACUGGAGGG	AGGAGGGGUGAGGGACUGGAGGG	-29.64	-23.60
C > A chr3:42845880	LUAD-5V8LT	HIGD1A	Lung	-	GGGACUCGGGCCGCGGGGCGGG	GGGACUCGGGCCGCGUGGCGGG	-101.87	-98.82
C > A chr4:75719847	LUAD-5V8LT	BTC	Lung	-	GGGUGUGGGUAUGGGUGUGGGGCAAUUUGGG	GGGUGUGGGUAUGGGUGUGUGGCAAUUUGGG	-155.85	-144.91
G > T chr6:53659864	LUAD-5V8LT	LRRC1	Lung	+	GGCGGGCGGGGGUACAGGGACGGGGCAGGG	GGCGGGCGGGGGUACAGGGACGUGGCAGGG	-158.65	-149.79
C > A chr8:144911228	LUAD-S01302	PUF60	Lung	-	GGGCGGGGCGGGGGCCUGGG	UGGCGGGGCGGGGGCCUGGG	-116.40	-111.10
C > T chr3:141944386	DO27813	GK5	Malignant Lymphoma	-	GGGUUGGGGCCCGGGUUGGG	GGGUUGGGGCCCGAGUUGGG	-58.80	-57.00

C > T chr18:60985950	DO27801	BCL2	Malignant Lymphoma	-	GGGCCGUGGGGUGGGAGCUGGG	GGGCCGUGGGGUGGAAGCUGGG	106.25	104.50
G > C chr22:23230078	DO27851	IGLL5	Malignant Lymphoma	+	GGGCCUGGGCUAGGGACAGGG	GGGCCUGGCCUAGGGACAGGG	-159.95	-157.30
G > A chr22:23230078	DO27855	IGLL5	Malignant Lymphoma	+	GGGCCUGGGCUAGGGACAGGG	GGGCCUGGACUAGGGACAGGG	-159.95	-154.50
C > T chr7:56119187	DO27827	PSPH	Malignant Lymphoma	-	GGGCAGGGCGUAGGGUGGG	GGGCAGGACGUAGGGUGGG	-249.99	-239.30
G > A chr3:45730840	TCGA-DA-A3F3	SACM1L	Melanoma	+	GGGCGGGGAAAGGGGUGGG	GGGCGGGGAAAGAGGUGGG	-94.48	-73.69
G > A chr7:127233863	TCGA-DA-A3F8	FSCN3	Melanoma	+	GGGGGGGAGGGCUGGG	GGGGAGGAGGGCUGGG	-98.49	-83.40
G > A chr8:22022672	TCGA-DA-A3F8	BMP1	Melanoma	+	GGAGGGAGGGAGGGAGGG	GGAGGGAGGGAGGGAGAG	-137.32	-106.14
G > A chr20:43595130	TCGA-EB-A24D	STK4	Melanoma	+	GGGAGGGUCUGCGGGGCGGG	AGGAGGGUCUGCGGGGCGGG	-36.60	-22.30
G > A chr7:99102730	TCGA-EE-A29B	ZKSCAN5	Melanoma	+	GGGUGGGCUGGGACUCGGG	GGGUGGGCUGAGACUCGGG	-90.49	-85.80
C > T chr2:75937669	TCGA-EE-A2A0	GCFC2	Melanoma	-	GGGCGGGAAGGGGCCGCGGG	GGGCGGAAAGGGGCCGCGGG	-111.50	-100.80
G > A chr8:124194467	TCGA-EE-A2GN	FAM83A	Melanoma	+	GGGCAGGGAGGGGUGGG	GGGCAGGGAGGGGUGAG	-646.42	-633.90

C > T chr17:36955814	TCGA-EE-A2GN	PIP4K2B	Melanoma	-	GGGUGGGAGGUAACGGGACGGG	GGGUGGAAGGUAACGGGACGGG	-295.37	-290.82
C > T chr12:111126954	TCGA-EE-A2M5	HVCN1	Melanoma	-	GGGCGGGACGGGCUGGG	GGGCGAGACGGGCUGGG	-82.32	-67.10
C > T chr2:148778353	TCGA-EE-A3JI	ORC4	Melanoma	-	GGGCGGGAGAGGGGCGGGGUGGG	GGGCGGAAGAGGGGCGGGGUGGG	-95.47	-85.74
C > T chr15:86338177	TCGA-ER-A19E	KLHL25	Melanoma	-	GGGCCGGGAGGCGGGGCGGG	GGGCCGGAAGGCGGGGCGGG	-102.20	-99.40
G > A chr10:31608106	TCGA-FS-A1ZD	ZEB1	Melanoma	+	GGGGGGGAAGGGGGAGGG	GGGGGAGAAGGGGGAGGG	-42.37	-24.37
C > T chr5:95297622	TCGA-FS-A1ZK	ELL2	Melanoma	-	GGGGCGGGGCGCGGGGCCGGAGGGUGGGGCGGG	GAGGCGGGGCGCGGGGCCGGAGGGUGGGGCGGG	-176.83	-171.70
C > A chr20:48729710	TCGA-GN-A262	UBE2V1	Melanoma	-	GGCCGGGGGACGGGUAAGGGGGGG	GGCCGGGGGACGGGUAAGUGGGGG	-122.17	-110.62
C > T chr4:89079865	TCGA-GN-A266	ABCG2	Melanoma	-	GGUUGGGGGAAGG	GGUUGGGGAAAGG	-177.02	-175.30
C > T chr5:134914827	TCGA-GN-A26C	CXCL14	Melanoma	-	GGGCGGGCGACGGGCGGGCUGGG	GGGCAGGCGACGGGCGGGCUGGG	-199.06	-189.89
C > T chr5:134914828	TCGA-GN-A26C	CXCL14	Melanoma	-	GGGCGGGCGACGGGCGGGCUGGG	GGGCGAGCGACGGGCGGGCUGGG	-199.06	-188.49
C > T chr17:78194181	TCGA-GN-A26C	SGSH	Melanoma	-	GGGGUCGGGGCAGGGGGCGGGG	GGGGUCGGGGCAGGAGGCGGGG	-56.57	-36.20

C > A chr10:27529738	DO46478	ACBD5	Ovarian	-	GGGCGGGGCUGUGGGGAGGGCACGGACUGACAGACGGACUCCGGCGGAAUGGGGG	GGGCGGGGCUGUGGGGAGGUCACGGACUGACAGACGGACUCCGGCGGAAUGGGGG	-97.30	-87.92
G > T chr19:34663401	DO46342	LSM14A	Ovarian	+	GGGCGGGAGGCUGGGGGAGGG	GGGCGGGAGGCUGGUGGAGGG	-112.95	-109.29

[a] RNA G4 sequence is predicted by RNAfold in the context of the full-length 5' UTR. Underlined G shows a guanine involved in the G-tetrad formation. Mutated nucleotide is depicted in red.

[b] ΔG (minimum free energy) is calculated by RNAfold for the entire 5' UTR.

[c] Coordinates are based on genomic reference sequence.

Supplementary Table 3. 5′ UTR G4 stabilising mutations

Mutation details				G4 sequence [a] (5′ → 3′)			ΔG (kcal/mol) [b]	
**Mutation Coordinate [c]**	**Patient's ID**	**Gene**	**Cancer**	**Strand**	**Wild-type**	**Mutant**	**Wild-type**	**Mutant**
G > C chr10:115613904	PD5935a	DCLRE1A	Breast	-	GGUGGGGGCGG	GGUGGGGGGGG	-245.19	-253.50
T > G chr1:236687199	PD5956a	LGALS8	Breast	+	GGGCGCGGGGAGGGUGGGG	GGGCGCGGGGAGGGGGGGG	-153.87	-158.90
A > G chr16:29985210	TCGA-A6-6781_strelka	TAOK2	Colon	+	GGGAGGAAGAGGGAGAGGGAGACCGGG	GGGAGGGAGAGGGAGAGGGAGACCGGG	-335.29	-344.98
A > C chr5:157002814	LUAD-S01356	ADAM19	Lung	-	GGGCUGGGAGCCGGGUGGGGAGG	GGGCUGGGAGCCGGGGGGGGAGG	-63.79	-66.00
A > C chr1:205782071	LUAD-S01404	SCL41A1	Lung	-	GGGGGUGUGGGGGGGCACGCCGGG	GGGGGGGUGGGGGGGCACGCCGGG	-312.35	-335.80
A > G chr21:34775413	LUAD-S01404	IFNGR2	Lung	+	GGGGGGGUCAGGAGGGGUGGG	GGGGGGGUCAGGGGGGGUGGG	-359.80	-373.37
A > C chr12:100378070	LUAD-S01405	ANKS1B	Lung	-	UGGGGAGGAGG	GGGGGAGGAGG	-184.40	-187.50
A > G chr17:55333994	LUAD-S01405	MSI2	Lung	+	GGGCGGGGGGGAGGAGG	GGGCGGGGGGGAGGGGG	-135.20	-154.30
T > G chr20:58179659	LUAD-S01405	PHACTR3	Lung	+	GGGGGUGGGGGGUGGGGUGGGGGG	GGGGGUGGGGGGGGGGGUGGGGGG	-157.02	-183.20

T > G chr20:58179659	DO32904	PHACTR3	Pancreatic	+	GGGGGUGGGGGGUGGGGUGGGGGG	GGGGGUGGGGGGGGGGGUGGGGGG	-157.02	-183.20
T > G chr20:58179664	DO46372	PHACTR3	Ovarian	+	GGGGGUGGGGGGUGGGGUGGGGGG	GGGGGUGGGGGGUGGGGGGGGGGG	-157.02	-174.89
G > C chr22:28197074	LUAD-S01405	MN1	Lung	-	GGGGCGGGGGGAGGG	GGGGGGGGGGGAGGG	-443.10	-452.00
T > G chr2:73144960	LUAD-S01405	EMX1	Lung	+	GGCGGGGGAGGUGAGGGGUGCGGGCGGG	GGCGGGGGAGGGGAGGGGUGCGGGCGGG	-214.20	-215.27
T > G chr1:110453297	TCGA-GN-A266	CSF1	Melanoma	+	GGGUGGGGGAGGGGAGGCGGGGG	GGGGGGGGGAGGGGAGGCGGGGG	-191.40	-192.27
T > G chr1:1260171	DO46342	CPTP	Ovarian	+	GGGGCGGUGGGCGGGGACGGGG	GGGGCGGGGGGCGGGGACGGGG	-199.17	-209.34
T > G chr1:53527802	DO46493	PODN	Ovarian	+	GGGGCGGGUGGGGAGUGGGGGAAGGGGG	GGGGCGGGGGGGGAGUGGGGGAAGGGGG	-83.54	-85.09
A > C chr19:45873690	DO46376	ERCC2	Ovarian	-	GGGGCGAGGGGAGGGUGAAGGGGUGGG	GGGGCGAGGGGAGGGGGAAGGGGUGGG	-187.88	-208.08
A > C chr19:47164380	DO46329	DACT3	Ovarian	-	GGGGCGGGGAGGUGGGACCGGGAGAGGGG	GGGGCGGGGAGGGGGGACCGGGAGAGGGG	-41.25	-51.03
T > G chr2:120770648	DO46372	EPB41L5	Ovarian	+	GGGCGGGAGGUCGGG	GGGCGGGAGGGCGGG	-83.72	-110.50
A > C chr7:123389082	DO46380	WASL	Ovarian	-	GGUUGGGGGAGG	GGGUGGGGGAGG	-143.99	-152.30

T > G chr9:135458144	DO46493	BARHL1	Ovarian	+	GGGGUUGGGGGUGGGUGG GG	GGGGUUGGGGGGGGGUGG GG	-83.07	-107.49

[a] RNA G4 sequence is predicted by RNAfold in the context of the full-length 5’ UTR. Underlined G shows a guanine involved in the G-tetrad formation. Mutated nucleotide is depicted in red.

[b] ΔG (minimum free energy) is calculated by RNAfold for the entire 5’ UTR.

[C] Coordinates are based on genomic reference sequence.

Supplementary Table 4. The most stable RNA G4 predicted by RNAfold and QGRS-Mapper in the 5'UTR of BCL2, CXCL14 and TAOK2

BCL2

RNAfold			
Wild-type		Mutant	
G4 sequence [a]	ΔG (kcal/mol)	G4 sequence	ΔG (kcal/mol)
GGGCCGUGGGGUGGGAGCUGGG	-11.05	GGGCCGUGGGGUGGAAGCUGGG	-1.20
**QGRS-Mapper**			
Wild-type		Mutant	
G4 sequence	G-score	G4 sequence	G-score
GGGCCGUGGGGUGGGAGCUGGG	40	GGGCCGUGGGGUGGAAGCUGG	19

[a] Underlined G shows a guanine involved in the G-tetrad formation predicted by each software. Mutated nucleotide is depicted in red.

CXCL14

RNAfold			
Wild-type		Mutant	
G4 sequence [a]	ΔG (kcal/mol)	G4 sequence	ΔG (kcal/mol)
GGGCGGGCGACGGGCGGGCUGGG	-19.37	GGGCAAGCGACGGGCGGGCUGGG	-9.64
**QGRS-Mapper**			
Wild-type		Mutant	
G4 sequence	G-score	G4 sequence	G-score
GGGCGGGCGACGGGCGGGCUGGG	39	GGGCAAGCGACGGGCGGGCUGGG	35

[a] Underlined G shows a guanine involved in the G-tetrad formation predicted by each software. Mutated nucleotide is depicted in red.

TAOK2

RNAfold			
Wild-type		Mutant	
G4 sequence [a]	ΔG (kcal/mol)	G4 sequence	ΔG (kcal/mol)
GGGAGGAAGAGGGAGAGGGAGACCGGG	-5.23	GGGAGGGAGAGGGAGAGGGAGACCGGG	-16.69
**QGRS-Mapper**			
Wild-type		Mutant	
G4 sequence	G-score	G4 sequence	G-score
GGGAGGAAGAGGGAGAGGGAGACCGGG	38	GGGAGGGAGAGGGAGAGGGAGACCGGG	40

[a] Underlined G shows a guanine involved in the G-tetrad formation predicted by each software. Mutated nucleotide is depicted in red.

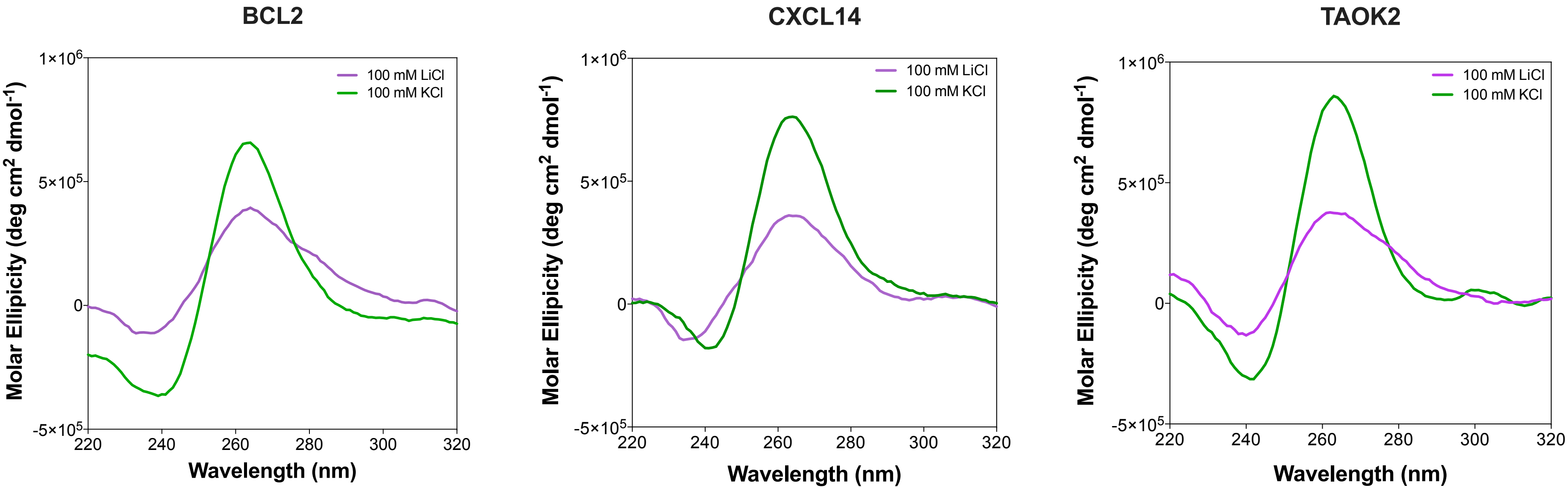

Supplementary Figure 1. CD spectra of *BCL2*, *CXCL14* and *TAOK2* wild-type G4 RNA oligos in 100 mM KCl or 100 mM LiCl (n ≥ 2).
